# Emerging Strategies to Improve the Design and Manufacturing of Biocompatible Therapeutic Materials

**DOI:** 10.3390/pharmaceutics15071938

**Published:** 2023-07-12

**Authors:** Guillermo Martínez, Juan Vázquez, Belén Begines, Ana Alcudia

**Affiliations:** 1 Departamento de Química Orgánica y Farmacéutica, Facultad de Farmacia, Universidad de Sevilla, 41012 Sevilla, Spain; gmartinez1@us.es; 2 Departamento de Química Orgánica, Facultad de Química, Universidad de Sevilla, 41012 Sevilla, Spain; cabello@us.es

**Keywords:** personalized medicine, controlled release, encapsulation, biodegradable materials, microparticles, nanoparticles, 3D printing, scaffolds, porous materials

## Abstract

Currently, the field of medicine is drastically advancing, mainly due to the progress in emerging areas such as nanomedicine, regenerative medicine, and personalized medicine. For example, the development of novel drug delivery systems in the form of nanoparticles is improving the liberation, absorption, distribution, metabolism, and excretion (LADME) properties of the derived formulations, with a consequent enhancement in the treatment efficacy, a reduction in the secondary effects, and an increase in compliance with the dosage guidelines. Additionally, the use of biocompatible scaffolds is translating into the possibility of regenerating biological tissues. Personalized medicine is also benefiting from the advantages offered by additive manufacturing. However, all these areas have in common the need to develop novel materials or composites that fulfill the requirements of each application. Therefore, the aim of this Special Issue was to identify novel materials/composites that have been developed with specific characteristics for the designed biomedical application.

## 1. Introduction

The development of novel materials or composites is of utmost importance in addressing and overcoming the numerous and complex challenges facing the field of medicine. The unique and specific requirements of different biomedical applications, ranging from drug delivery systems to tissue regeneration and personalized medical devices, demand materials that can effectively meet these varying needs. As such, the primary goal of this Special Issue is to showcase the latest advancements and research in the development of such materials or composites.

In addition to the challenges posed by the development of new materials, their safety and effectiveness for use in medical applications must equally be ensured. This necessitates the use of advanced characterization techniques to gain a thorough understanding of the properties and behavior of these materials in biological environments. Additionally, regulatory compliance is an essential aspect to ensure that these materials meet the standards for use in medical applications.

Overall, the emergence of innovative areas such as nanomedicine, regenerative medicine, and personalized medicine has revolutionized how treatments are delivered, leading to substantially improved patient outcomes. Nevertheless, much remains to be achieved in the field of medicine, and the development of novel materials or composites is an indispensable step in overcoming these persistent challenges. Through this Special Issue, researchers and scientists can present their latest research and ideas in this field, thereby providing a platform for future innovations in medicine.

## 2. The Use of Metals for Antibacterial Applications

Hou et al. [1] developed nano-zinc-oxide particles as an example of nanometer-sized metallic entities exhibiting antibacterial and neurotoxicity behavior. They were tested against *Escherichia coli* (*E. coli*) and *Klebsiella pneumoniae* (*K. pneumonia*) microorganisms; additional nontoxic effects toward HMC-3 human normal brain microglia cells were reported, along with a potential cytotoxic effect on the LN-18 human brain glioblastoma cell line. The green synthesis methodology is based on a method applying an aqueous zinc nitrate salt and *Perilla frutescens* crude protein as protecting and reducing agents, respectively. The characterization included UV–visible spectrophotometry, X-ray diffraction, X-ray photoelectron spectroscopy, Fourier transform infrared spectroscopy, scanning electron microscopy, energy-dispersive X-ray spectroscopy, and high-resolution transmission electron microscopy. All data indicated a potential application of these nanoparticles as antibacterial and anticancer drugs for glioblastoma prevention.

In a similar application, silver complexes bearing amino-acid-derived N-heterocyclic carbene with interesting antibacterial properties were synthetized with different functionalizations by Sánchez et al. [2]. Complexes {Ag[NHC^Mes,R^]}_n_ (R = H, Me, ^i^Pr, ^i^Bu) were prepared via the treatment of imidazolium precursor compounds [Im^Mes,R^] (2-(3-mesityl-1H-imidazol-3-ium-1-yl)acetate, (S)-2-*alkyl*(3-mesityl-1H-imidazol-3-ium-1-yl)acetate, and (R)-2-methyl(3-mesityl-1H-imidazol-3-ium-1-yl)acetate, with Ag_2_O using optimal conditions. Analytical, spectroscopic (IR, ^1^H, and ^13^C NMR and polarimetry), and X-ray methods were used to find that in the solid state, a one-dimensional coordination was produced, bearing a silver(I) cation bonded to the carbene ligand as well as to a carboxylate group. The antimicrobial behavior of these silver complexes was evaluated against Gram-negative bacteria *E. coli* and *Pseudomonas aeruginosa* (*P. aeruginosa*) to determine the MIC and MBC values and reveal the structure–antimicrobial effect relationship. In conclusion, antimicrobial activity decreases when the steric properties of the R alkyl group in {Ag[NHC^Mes,R^]}_n_ strengthen.

## 3. Biodegradable Polymers as Innovative Tools for Biomedical Applications

Lamparelli et al. [3] described the synthesis and characterization of a new composite biopolymer scaffold, which is based on equine type I collagen and hyaluronic acid. The biomimetic structure was created using a reaction in the heterogeneous phase and was evaluated for its chemical, physical, and cytotoxicity properties using human-derived lymphocytes and chondrocytes. The successful reticulation of hyaluronic acid within the collagen structure was confirmed by FT-IR data, and TGA and DSC characterizations revealed the different thermal stabilities of cross-linked scaffolds. The results of SEM analysis suggested a highly porous structure with open and interconnected void areas that are suitable for hosting cells. The composite scaffold exhibited higher endurance with collagenase than with collagen alone, and its mechanical behavior showed excellent shape memory, especially when hydrated. The Young’s modulus was also considerably improved. The results of in vitro cytotoxicity testing confirmed that the scaffold was safe with human lymphocytes, and the gene expression profiles of chondrocytes showed excellent results.

To create personalized drug delivery systems with customized release rates, a series of bio-nanocomposites composed of shark gelatin hydrogels and PLA nanoparticles with different nanostructures was designed by Moya-López et al. [4]. The systems were developed considering the desired customization of drug release, viscoelastic properties for ease of storage and local administration, biocompatibility, and cell growth capability. The hydrogel matrix enabled the direct thermal conversion of the trapped nanostructures, avoiding the agglomeration of nanoparticles that could reduce their therapeutic effect. Two antitumoral compounds, doxorubicin and dasatinib, were successfully formulated in the nanoparticles to demonstrate the loading versatility of the drug delivery system. The bio-nanocomposites were characterized via several techniques such as SEM, DLS, Raman, DSC, SAXS/WAXS, and rheology. Their reversible sol–gel transition upon thermal treatment during the drug delivery system preparation and thermal annealing steps was also assessed. The drug delivery system’s local applicability was validated through the syringe test, which is used to evaluates storage capability and flow properties at simulated physiological conditions. Finally, the drug release profiles of doxorubicin from both the PLA nanoparticles and bio-nanocomposites were analyzed.

## 4. Use of Superficially Modified Porous Metals or Metal Alloys to Improve Osseointegration

In recent decades, the relevance of dental implantology has increased due to the growing world population and longer life expectancy, with the concomitant heightened focus on physical appearance. Consequently, various engineering strategies have been investigated to improve the survival rate of dental implants, including material composition, geometry, and surrounding interface tissues. Although several implant surface modifications are being applied in commercial dental prostheses, surface coatings have received special interest because they can be tailored to efficiently enhance osseointegration while minimizing bacterial-related infections and the associated risks of peri-implantitis. Furthermore, the use of biomaterials to replace teeth has emphasized the need for reliable analytical methods to assess the therapeutic benefits of implants. The literature review presented by Accioni et al. [5] cover the state-of-the-art strategies for surface modification or coating and analytical methodologies to increase the survival rate of tooth restoration.

Accioni et al. [6] also developed a novel microtechnology that uses polycaprolactone microspheres to adhere to porous titanium implant models produced by the spacer holder technique. This allowed for a custom balance of biomechanical and biofunctional properties. The microparticles were fabricated using a double emulsion solvent evaporation technique and were loaded with the antibacterial therapeutic agent, rose Bengal. These microspheres were infiltrated into a porous titanium substrate and sintered at 60 °C for 1 h, resulting in a convenient prophylactic network. The sintered polymeric microparticles played a key role in controlling the drug dissolution rate and promoting early healing by improving the wettability of the porous titanium substrate, which facilitated calcium phosphate nucleation. This joint technology is a suitable prophylactic tool to prevent early stage infections and late-stage osseointegration problems. By combining the benefits of porous dental implants, biopolymers, and microtechnology, this approach offers a promising solution to some of the challenges that currently limit the success of dental implant procedures.

Mechanical-based surface modification techniques are inefficient for implants with complex geometries produced by additive manufacturing (AM). As a result, plasma immersion ion implantation (PIII) is the most effective alternative as it creates nanotopography even in complex structures. Iatecola et al. [7] investigated the osseointegration results of the three conditions of additively manufactured Co–Cr–Mo alloy: (i) as-built, (ii) after PIII, and (iii) coated with titanium (Ti) followed by PIII. The metallic samples were designed with a solid half and a porous half to observe the bone ingrowth for different surfaces. All conditions showed cortical bone formation; however, the titanium-coated sample had the best biomechanical results due to the higher bone ingrowth percentage, with nearly all medullary canals filled with neoformed bone and the pores of the implant filled and surrounded by bone ingrowth. In conclusion, metal alloys produced for AM are biocompatible and stimulate bone neoformation, particularly when using Co–28Cr–6Mo alloy with a Ti-coated surface that is nanostructured and anodized by PIII. This technology was found to increase the osseointegration capacity of implants.

## 5. Fabrication Aspects in the Biomedical Field

Orodispersible films (ODFs) are a type of oral drug delivery dosage form that is thin, rapidly disintegrates, and is suitable for pediatric and geriatric patients. These films can be produced using various techniques, but the most commonly used method is the solvent casting method (SCM), which is simple and suitable for industrial production. Other methods for producing ODFs include extrusion, printing, electrospinning, and a combination of these technologies (e.g., SCM + printing). Gupta et al. [8] provide a comprehensive overview of patented technologies used in the last 20 years to fabricate ODFs. The review concludes that SCM is the most popular method, whereas electrospinning is a more recent and upcoming method for creating ODFs. The review also discusses other patent-protected technologies, particularly in the areas of printing (2D or 3D), extrusion (ram or hot-melt extrusion), electrospinning, or a combination of these methods.

Finally, the manufacture of advanced therapy medicinal products (ATMPs) involves complying with the strict guidelines of a pharmaceutical quality system, adhering to good manufacturing practices (GMPs), and using a cleanroom with stringent environmental conditions (Class A work area and Class B environment), all of which increase the cost of ATMP production. Furthermore, the limited use of many of these therapeutic products for a few patients and the single batch per manufacturing unit result in unprofitability. To address these concerns, ATMPs can be produced in a scaled-down system such as an isolator, which provides a highly controlled environment isolated from the external environment and allows for placement of the facility in a less stringent and more cost-effective Class D environment. Berisa-Prado et al. [9] demonstrate that bioengineered corneal epithelium can be manufactured in an isolator at an affordable cost for patients, complying with GMP guidelines and safety assurance standards. This small-scale ultra-clean working environment offers a promising solution to the high costs associated with conventional cleanroom ATMP production.

## 6. Conclusions

In conclusion, we need to prioritize the development of novel materials, composites, and techniques to meet the diverse and unique demands of various biomedical applications, which encompass microbial resistance, drug delivery systems, tissue regeneration, and personalized medical devices. The Special Issue highlights the latest advancements and research in the development of these critical materials, composites, or technologies. However, ensuring their safety and effectiveness for medical applications remains a daunting challenge, necessitating the use of advanced characterization techniques and regulatory compliance.

The manuscripts published in this Special Issue address different aspects of the design and manufacturing of biocompatible therapeutic materials. In this sense, two manuscripts describe the use of metals for antibacterial applications, two more describe the application of biodegradable polymers as innovative tools for biomedical applications, another three propose the use of superficially modified porous metals or metal alloys to improve osseointegration, and the last two focus on the fabrication aspects to be considered in the biomedical field.

In summary, despite these challenges, emerging fields such as nanomedicine, regenerative medicine, and personalized medicine have revolutionized how treatments are delivered, leading to improved patient outcomes. This Special Issue offered an excellent opportunity for researchers and scientists to present their latest findings, research, and innovative ideas, paving the way for future developments in medicine.
